# MicroRNA-155 regulates monocyte chemokine and chemokine receptor expression in Rheumatoid Arthritis

**DOI:** 10.1093/rheumatology/kew272

**Published:** 2016-07-13

**Authors:** Aziza Elmesmari, Alasdair R. Fraser, Claire Wood, Derek Gilchrist, Diane Vaughan, Lynn Stewart, Charles McSharry, Iain B. McInnes, Mariola Kurowska-Stolarska

**Affiliations:** ^1^Institute of Infection, Immunity and Inflammation, University of Glasgow, Glasgow, UK; ^2^Benghazi Medical Center, Medical School, Benghazi University, Benghazi, Libya; ^3^Development and Innovation, Scottish National Blood Transfusion Service, Research, Edinburgh, UK

**Keywords:** microRNA-155, monocyte, chemokines, rheumatoid arthritis, disease activity

## Abstract

**Objective.** To test the hypothesis that miR-155 regulates monocyte migratory potential via modulation of chemokine and chemokine receptor expression in RA, and thereby is associated with disease activity.

**Methods.** The miR-155 copy-numbers in monocytes from peripheral blood (PB) of healthy (n = 22), RA (n = 24) and RA SF (n = 11) were assessed by real time-PCR using synthetic miR-155 as a quantitative standard. To evaluate the functional impact of miR-155, human monocytes were transfected with control or miR-155 mimic, and the effect on transcript levels, and production of chemokines was evaluated by Taqman low-density arrays and multiplex assays. A comparative study evaluated constitutive chemokine receptor expression in miR-155^−/−^ and wild-type murine (CD115 ^+^ Ly6C ^+^ Ly6G^−^) monocytes.

**Results.** Compared with healthy monocytes, the miR-155 copy-number was higher in RA, peripheral blood (PB) and SF monocytes (PB P < 0.01, and SF P < 0.0001). The miR-155 copy-number in RA PB monocytes was higher in ACPA-positive compared with ACPA-negative patients (P = 0.033) and correlated (95% CI) with DAS28 (ESR), R = 0.728 (0.460, 0.874), and with tender, R = 0.631 (0.306, 0.824) and swollen, R = 0.503 (0.125, 0.753) joint counts. Enforced-expression of miR-155 in RA monocytes stimulated the production of CCL3, CCL4, CCL5 and CCL8; upregulated CCR7 expression; and downregulated CCR2. Conversely, miR155^−/−^ monocytes showed downregulated CCR7 and upregulated CCR2 expression.

**Conclusion.** Given the observed correlations with disease activity, these data provide strong evidence that miR-155 can contribute to RA pathogenesis by regulating chemokine production and pro-inflammatory chemokine receptor expression, thereby promoting inflammatory cell recruitment and retention in the RA synovium.

Rheumatology key messages

- The copy number of miR-155 in blood monocytes correlates strongly with clinical markers of disease activity.
- miR-155 can contribute to RA pathogenesis by regulating chemokine production and pro-inflammatory chemokine receptor expression.
- Induction of chemokine production and down-regulation of chemokine receptor may contribute to monocyte retention at inflammation sites in RA.

## Introduction

A critical pathological feature of RA is the accumulation of monocytes/macrophages in the synovial tissue [1], where they serve as predominant cytokine-producing effector cells [2–4]. The recruitment of monocytes from the blood is an important step in disease progression and is mediated by locally produced chemokines [1]. An improved understanding of the mechanisms regulating the recruitment, migration and retention of these cells in RA could facilitate development of novel biomarkers and therapeutics.

miRNAs are a recently discovered class of post-transcriptional regulators that induce target mRNA degradation or translation inhibition [5]. Of particular relevance in the context of joint inflammation in RA is miR-155. Its expression is upregulated in RA synovial monocytes/macrophages and fibroblasts [6, 7]. Its overexpression in macrophages triggers the production of pro-inflammatory mediators, including TNF-α [6].

Less is known about the expression and function of miR-155 in RA peripheral blood (PB) monocytes. PB monocytes serve as precursors for a proportion of synovial monocytes/macrophages and conceivably circulate in a primed state ready for recruitment. Epigenetic regulation of their ability to respond to chemokines might therefore contribute to pathogenesis. We hypothesized that blood and synovial monocytes from RA patients exhibit miR-155-dependent chemokine and chemokine receptor regulation that determines their potential for recruitment to the joint. This hypothesis was tested by quantifying the copy-number of miR-155, which then allowed direct comparison between blood and synovial monocytes; we then used this knowledge to investigate the role of miR-155 in the regulation of chemokine and chemokine receptors expression as measures of monocyte migratory potential.

We report here that compared with healthy blood CD14^+ ^monocytes, CD14^+ ^monocytes from RA patients have a higher copy-number of miR-155, and this was correlated with RA disease activity. Furthermore, the miR-155 copy-number was higher again in RA SF CD14^+ ^cells. Functionally, we discovered that miR-155 in RA monocytes is induced more robustly by inflammatory challenge than control monocytes, and that miR-155 enhanced chemokine production and downregulated the expression of the pro-inflammatory chemokine receptor, CCR2. Commensurate with this, CCR2 was up regulated in miR155^−^^/^^−^ monocytes. These data suggest that an elevated miR-155 copy-number in monocytes contributes to inflammatory cell recruitment (by increasing local chemokine production) and to the retention of cells in RA joints by reducing their migratory chemokine receptors.

## Materials and methods

### Patients and healthy donors

PB samples were obtained from RA patients at Glasgow Rheumatology clinics and from age- and gender-matched healthy control (HC) subjects. RA patients met the 2010 ACR/EULAR diagnostic criteria. Demographic, clinical and laboratory information is detailed in supplementary Table S1, available at *Rheumatology* Online. SF samples were collected from RA patients at various routine outpatient Rheumatology Clinics (Glasgow, UK). Demographic, clinical and laboratory information is detailed in supplementary Table S2, available at *Rheumatology* Online. This study was approved by the West of Scotland Research Ethics Service and all subjects provided signed informed consent.

### Human cell culture

#### Monocytes

CD14^+ ^monocytes from 50 ml PB from healthy donors (n = 22) and RA patients (n = 24), and from RA SF (n = 11; ∼20–25 ml collected) were isolated using CD14^+ ^micro-beads (Miltenyi) and an Auto-MACS separator according to the manufacturer’s protocol. This resulted in an average of 10.4 (3.5) and 8.8 (2.5) of PB CD14+ cells per healthy and RA donor, respectively. We obtained between 6 and 11 × 10^6^ SF CD14^+ ^cells. The purity of monocytes was evaluated by flow cytometry (supplementary Fig. S1 and Table S2, available at *Rheumatology* Online). PB CD14^+ ^monocytes (0.35 × 10^6^ per well of a 24-well plate) were either transfected with miR-155 (functionally mature miR-155 mimic), control miR mimic or fluorescent control mimic (CM) Dy547 to demonstrate transfection efficiency (at 20 nM; Dharmacon), using the N-TER transfection reagent (Sigma), or were left untransfected as a control. After 48 h, the cells and supernatant were collected. In some cultures, monocytes from healthy donors were incubated with RA SF (n = 3) and expression of miR-155 quantified. The assessment of chemokine production and mRNA expression, and chemokine receptor mRNA expression was tested only in cultures where the transfection efficiency was >60% and showed an increase in miR-155 expression (supplementary Fig. S2, available at *Rheumatology* Online). This occurred in 15 HCs and in 16 RA patients. These are listed in supplementary Table S3. The details of this subgroup did not differ from the main sample population (supplementary Table S1, available at *Rheumatology* Online) and they were therefore considered as representative. In addition, PB CD14^+ ^monocytes of HCs and RA patients were cultured alone (HC n = 22, RA in remission n = 5, active RA n = 19) or in the presence of different doses of lipopolysaccharide (LPS) (2 ng/ml; HC n = 18, RA in remission n = 5, active RA n = 17) or (10 ng/ml; HC n = 9, RA in remission n = 0, active RA n = 16) for 24 h to determine the effect of inflammatory challenge on miR-155 expression.

#### T cell—macrophage co-cultures

CD4^+ ^cells were isolated from HCs (n = 6) using CD4 microbeads (Militenyi) and the memory T cell subpopulation expanded and activated by incubation with IL-15 (25 ng/ml), TNF (25 ng/ml) and IL-6 (100 ng/ml) as described before [8]. CD14^+ ^cells from the same donors were differentiated to macrophages by incubation with M-CSF (50 ng/ml). After 6 days T cells were added to monocyte-derived macrophages at a ratio of 8:1 for 24 h as described, and *in situ* hybridization for miR-155 in macrophages was performed [8].

#### Mouse monocytes

Bone marrow monocytes were FACS-sorted from wild-type and miR-155^−^^/^^−^ mice based on the expression of CD11b, CD115, Ly6C and lack of Ly6G as described [9]. Detailed information and the flow cytometry gating strategy are provided in supplementary Fig. S6, available at *Rheumatology* Online.

#### RNA isolation and cDNA synthesis

Total RNA was extracted using miRNeasy mini Kit (Qiagen). cDNA was transcribed from RNA using miScript Reverse Transcription Kit (Qiagen) according to the manufacturer’s instruction.

#### Quantifying miR-155 copy-number

Known copy numbers of synthetic miR-155 mimic (Dharmacon) and plasmid with cloned housekeeping control RNU1A were used as standards. To obtain a standard curve, these were diluted in serial 10-fold dilutions in nuclease-free water to generate a range of standards from 1 × 10^3^ to 1 × 10^9^ copies. Each of these standards was used alongside cDNA from the samples in qPCR (miScript Sybr Green PCR kit; Qiagen) with specific human miR-155 and RNU1A primers (Qiagen). Based on the standard curves the copy number of miR-155 and RNU1A in the samples were calculated and data were presented as the copy-number of miR-155 per 1 000 000 copies of RNU1A. In some experiments, the expression of miR-155 is presented as a relative value: 2^−^^Δ^ ^*C*^_t_ where Δ*C*_t_ = Cycle threshold for RNU1A minus Ct for miR-155.

#### Luminex

The concentration of chemokines and cytokines in CD14^+ ^culture supernatants was quantified by immuno-fluorescence assay using the Multiplex Kit panel I and II (Millipore UK) on a Bio-Plex platform (Bio-Rad). The plates were designed to measure TNF-α, IL-1β, IL-10, CCL2/MCP-1, CCL3/MIP-1α, CCL4/MIP-1β, CCL5/RANTES, CCL7/MCP-3, CCL8/MCP-2, CCL13/MCP4, CCL17/TARC, CCL19/MIP-3β, CCL20/MIP-3α, CCL21/6CKINE, CCL22/MDC, CXCL1/GRO, CXCL5/ENA-78, CXCL7/NAP-2, CXCL8/IL-8, CXCL9/MIG, CXCL10/IP-10, CXCL11/I-TAC, CXCL12/SDF-1, CX_3_CL1/Fractalkine, lymphotactin and vascular endothelial growth factor A (VEGFA).

#### Taqman low-density arrays for chemokine and chemokine receptor expression

RNA (550 ng) was reverse-transcribed with High Capacity RNA-to cDNA Kit (Applied Biosystems) following the manufacturer’s guidelines. Custom-designed TaqMan low-density arrays plates containing specific primers and probes for 14 human chemokines/cytokines (CCL2–5, 7, 8; CCL22; CXCL1; TNF-α; IL-1β; IL-6; IL-8; IL-10; and VEGF) or for 16 chemokine receptors (CCR1–10; CXCR1, 2, 4; XCR1; CX_3_CR1; and CMKLR1); as well as for 17 mouse chemokine receptors (CCR1–3, CCR5–10, CXCR2–6, CX_3_CR1 and CXCR1) were used with TaqMan PCR Master Mix, No AmpErase (ABI Ltd) and were run on a 7900HT TaqMan reader. Δ*C*_t_ values were calculated using 18S rRNA as an endogenous control, and ΔΔ*C*_t_ values were calculated by comparison with the mean Δ*C*_t_ of control samples (CM or wild-type monocytes).

#### FISH combining fluorescent immunohistochemistry

*In Situ* hybridization combined with immunohistochemistry for CD68 was performed using a lock nucleic acid (LNA) microRNA *in situ* hybridization Optimization Kit, LNA 5′- and 3′-digoxigenin-labelled scramble (GTGTAACACGTCTATACGCCCA) and miR-155–specific (TATCACGATTAGCATTAA) probes (all from Exiqon), as described previously [6].

### Statistical analysis

The data were analysed by Graph Pad Prism version 5.0 and Minitab software. Intergroup differences were tested by t-test or by Mann–Whitney U test, depending on data distribution or after analysis of variance or Kruskal–Wallis tests for multiple groups. Data was expressed as means (s.d.) or median and interquartile range. Correlation between variables was tested using the two-tailed Pearson Correlation Coefficient with 95% CI. A P-value  < 0.05 was considered significant.

## Results

### 

#### MiR-155 copy-number in RA PB monocytes was elevated over normal donors and correlated with disease activity

**Fig. 1:**
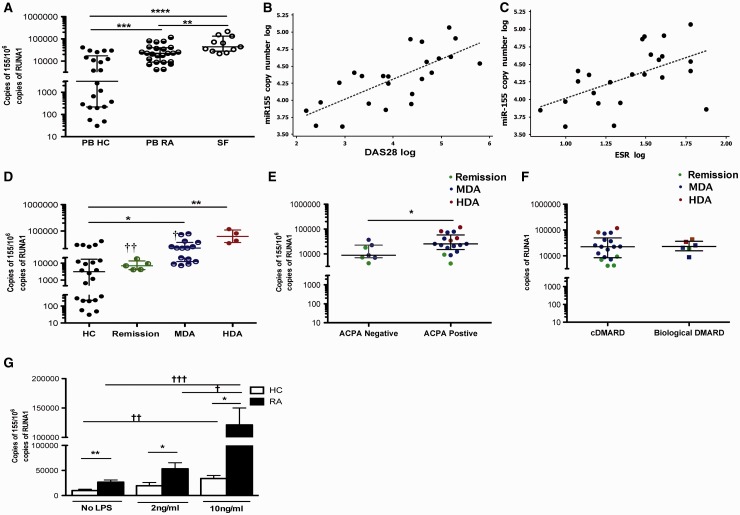
Copy-number of miR-155 in RA monocytes (**A**) miR-155 in CD14+ of RA peripheral blood (PB) (n = 24), SF (n = 11) and healthy controls (HCs; n = 22). (**B** and **C**) Correlations between miR-155 and clinical parameters. (**D**) miR-155 in RA with clinical remission, moderate (MDA) and high disease activity (HDA). (**E**) miR-155 in ACPA-positive *vs* ACPA-negative patients. (**F**) miR-155 in conventional (c) *vs* biological disease-modifying antirheumatic drugs (DMARDs) treated RA. (**G**) miR-155 in PB CD14+ of HCs (n = 9–22) and RA patients (n = 16–24) cultured with LPS for 24 h. *HC *vs* RA (*P ≤ 0.05, **P ≤ 0.005 and ***P ≤ 0.0005); ^†^stimulation *vs* control conditions (^†^P ≤ 0.05, ^††^P ≤ 0.005 and ^†††^P ≤ 0.0005). Data are presented as median (interquartile range).

We, and others, have shown an increased relative expression of miR-155 in RA peripheral blood mononuclear cells (PBMCs) [10], synovial biopsies, synovial fibroblasts and SF-derived CD14^+ ^cells [6, 7]. However, the quantitative expression of miR-155 in purified blood CD14^+ ^monocytes and its relation to disease activity has not been elucidated. Therefore, we developed a method to calculate the copy-numbers of miR-155 transcripts, which provides a more accurate foundation compared with relative expression methods for biomarker studies. The copy-numbers of miR-155 transcripts in purified PB (P < 0.009) and SF CD14^+ ^monocytes (P < 0.0001) of RA patients were significantly higher than the CD14^+ ^monocytes of HCs (Fig. 1A).

To investigate the relationship between miR-155 in purified blood monocytes and disease parameters, the miR-155 copy-number in PB CD14^+ ^was correlated against a variety of clinical indices and laboratory biomarkers. The miR-155 copy-number correlated with DAS28 (based on ESR) [Pearson’s R (95% CI) R = 0.728 (0.460, 0.874), P < 0.001 (Fig. 1B)] and with ESR [R = 0.546 (0.183, 0.778), P = 0.006 (Fig. 1C)]. The miR-155 copy-number in PB CD14^+ ^cells positively correlated with total joint [R = 0.631 (0.306, 0.824), P < 0.001] and swollen joint [R = 0.503 (0.125, 0.753), P = 0.012] counts (Table 1). Patients in remission showed comparable levels of miR-155 to HCs (Fig. 1D), while patients with high disease activity demonstrated the highest copy-number compared with those with moderate disease activity (P = 0.018) or remission state (P = 0.015) and HCs (P = 0.0025). There was an increased expression of miR-155 copy-number in monocytes from patients positive for ACPA compared with ACPA-negative patients (P = 0.033) (Fig. 1E). This was associated with a trend for higher DAS28 in the ACPA-positive group [4.25 (0.93) *vs* 3.47 (0.98); P = 0.07]. We found no difference in miR-155 copy-number in PB CD14^+ ^cells between patients treated with conventional DMARDs or biologics (Fig. 1F). There was no correlation between the miR-155 copy-number in PB CD14^+ ^cells and the patient’s age or disease duration (Table 1). Together these data suggest that an elevated miR-155 is present in RA PB monocytes prior to establishment at sites of inflammation. To investigate whether a high copy number of miR-155 in SF CD14^+ ^cells is associated with the disease activity in these patients, we did a correlation analysis comparing copy number with DAS28 (ESR). These patients had a moderate DAS28 (ESR) [mean (s.d.) of 3.8 (0.6)] (supplementary Table S2, available at *Rheumatology* Online). This did not correlate significantly with the miR-155 copy number in SF monocytes (r = 0.431, P = 0.186), suggesting that additional local factors present at the site of inflammation effected an extremely high expression of miR-155 in SF cells.

**Table 1:** Correlation of miR-155 copy numbers with a variety of demographic and clinical indices

	miR-155 copy no.	Age	DD	ESR	CRP	TJS	SJS
Age	−0.239						
	(−0.58, 0.18)						
	0.260						
DD	−0.064	0.464					
	(−0.45, 0.34)	(0.07, 0.73)					
	0.766	0.022					
ESR	0.546	−0.136	−0.108				
	(0.18, 0.77)	(−0.51, 0.28)	(−0.49, 0.30)				
	0.006	0.526	0.615				
CRP	0.186	−0.169	−0.095	0.672			
	(−0.23, 0.54)	(−0.53, 0.25)	(−0.48, 0.32)	(0.36, 0.84)			
	0.384	0.429	0.659	0.000			
TJS	0.631	−0.035	0.127	0.261	0.197		
	(0.30, 0.82)	(−0.43, 0.37)	(−0.29, 0.50)	(−0.15, 0.60)	(−0.22, 0.55)		
	0.001	0.872	0.555	0.218	0.356		
SJS	0.503	−0.232	−0.301	0.565	0.472	0.529	
	(0.12, 0.75)	(−0.58, 0.18)	(−0.62, 0.11)	(0.21, 0.78)	(0.08, 0.73)	(0.16, 0.76)	
	0.012	0.276	0.152	0.004	0.020		
DAS28	0.728	−0.193	−0.055	0.759	0.600	0.781	0.803
	(0.46, 0.87)	(−0.55, 0.22)	(−0.44, 0.35)	(0.51, 0.88)	(0.26, 0.80)	(0.55, 0.90)	(0.59, 0.93)
	0.000	0.366	0.797	0.000	0.002	0.000	0.000

R-values are on top, with 95% CIs below and corresponding P-values in the subsequent line. DD: disease duration; TJS: total joint score; SJS: swelling joint score.

#### Active RA blood monocytes expressed higher copy-numbers of miR-155 upon LPS stimulation than healthy monocytes

Next, we investigated whether RA monocytes express disproportionately elevated copy-numbers of miR-155 upon inflammatory stimulus. We quantified the copy-number of miR-155 in monocytes from HC subjects and RA patients before and after treatment with different doses of LPS (2 and 10 ng/ml) (Fig. 1G). The miR-155 copy-number was constitutively increased in RA monocytes. The addition of LPS dose-dependently increased the copy-number of miR-155 in CD14^+ ^monocytes, and this was exacerbated in RA patients (Fig. 1G). Upon stratification of RA patients into RA in remission and active RA, the constitutive miR-155 expression in RA patients in remission was similar to that of HC subjects, and responded to the similarly low-dose LPS found in HC subjects (supplementary Fig. S3, available at *Rheumatology* Online). This suggested that RA monocytes were epigenetically programmed at the periphery; with blood monocytes from active RA primed to robustly increase miR-155 expression in response to re-stimulation.

#### RA SF and a direct contact with T cells increased miR-155 expression

**Fig. 2:**
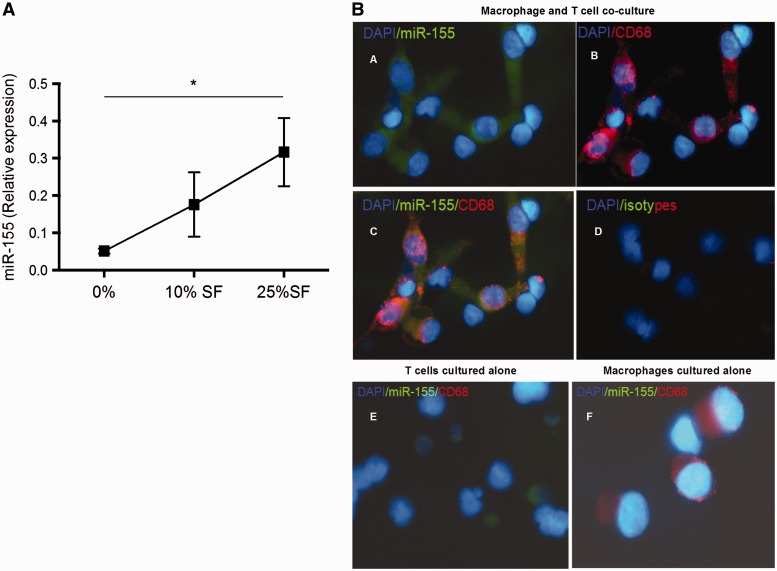
miR-155 was upregulated by RA synovial fluid and co-culture with autologous T cells (**A**) Peripheral blood (PB) monocytes were incubated with RA SF (n = 3) for 24 h and expression of miR-155 evaluated by relative expression to RNU1A. One-way analysis of variance tests, *P ≤ 0.05. (**B**) FISH for miR-155 (green) with immunohistochemistry staining for cell nuclei (blue) and macrophage marker (CD68^+^; red) were performed on macrophage–T cell co-cultures. (a–d) Macrophage and T cell co-cultures; (e) T cells alone. (f) Macrophages alone. Orange colour indicates double**-**positive CD68 and miR-155 cells.

To investigate which soluble and cellular factors upregulate miR-155 in monocytes in the RA synovial compartment, healthy blood monocytes were cultured with RA SFs, or monocyte-derived macrophages co-cultured with autologous activated memory T cells. Stimulation with SF or contact with activated memory T cells upregulated miR-155 transcript expression in these monocytes/macrophages (Fig. 2). These data indicate that the RA synovial environment (mediators or cells) may be responsible for an additional increase in the expression of miR-155 in RA SF CD14^+ ^as compared with blood CD14^+^.

#### miR-155 increased pro-inflammatory chemokine production by monocytes

**Fig. 3:**
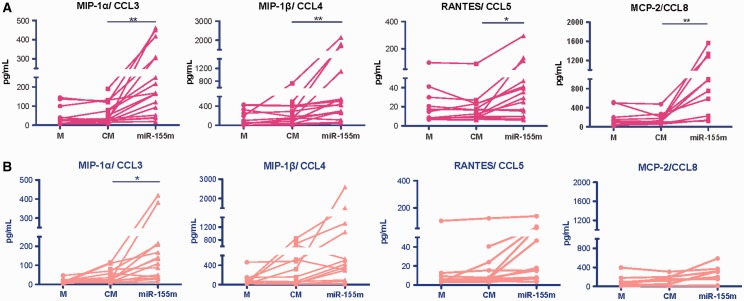
*In vitro* chemokine production in response to enforced expression of miR-155 Spontaneous 48 h *in vitro* chemokine production by peripheral blood (PB) CD14^+^ monocytes from (**A**) RA patients (n = 16) and (**B**) healthy controls (n = 15) after transfection with miR-155 mimic (miR-155m) or control mimic (CM), or by untransfected monocytes (M). Cells were tested in triplicate. Individual values are shown. Comparisons were analysed using paired *t*-test and *P ≤ 0.05 and **P ≤ 0.005.

miR-155 is a master regulator of cytokine production by human monocytes and macrophages, and its induced expression mimics pro-inflammatory activation of cells [6, 7]. Given the differential expression of miR-155 in RA monocytes from PB and synovial compartments, we investigated the contribution of miR-155 to epigenetic regulation of the chemokines and chemokine receptors that govern migration of monocytes from blood to sites of inflammation. To examine the role of miR-155 in chemokine production *in vitro*, we replicated the high expression levels of miR-155 in synovial CD14^+ ^by transfecting blood CD14^+ ^monocytes of HCs and RA patients with a functional miR-155 mimic (miR-155m) or CM and assessed the levels of chemokine production and mRNA expression by multiplex ELISA (14 chemokines; n = 15 healthy, n = 16 RA) and TaqMan low-density array (22 chemokines; n = 8 for both HCs and RA) assays, respectively. Enforced expression of miR-155 in CD14^+ ^blood monocytes from RA and healthy subjects stimulated the production of TNF-α (supplementary Fig. S4, available at *Rheumatology* Online) consistent with previous observations [6, 11], but in addition, stimulated the production of the chemokines CCL3, CCL4, CCL5 and CCL8 in RA and CCL3 in healthy monocytes compared with controls transfected with CM (Fig. 3). The supernatant concentrations of CCL2, CCL7, CCL21, CCL22, CXCL1, CXCL5, CXCL7, CXCL8, CXCL10 and CX_3_CL1 were low or unchanged, the latter suggesting that miR-155 did not regulate their expression (data not shown).

Next, we investigated mRNA transcript levels of CCL3, CCL4, CCL5, CCL8 and TNF-α. Consistent with protein expression (Fig. 3), mRNA transcript levels for these chemokines were upregulated in both RA and healthy monocytes transfected with miR-155 (supplementary Fig. S5, available at *Rheumatology* Online). These data suggest that miR-155 supports pro-inflammatory chemokine production.

#### miR-155 regulated chemokine receptor expression in monocytes

**Fig. 4:**
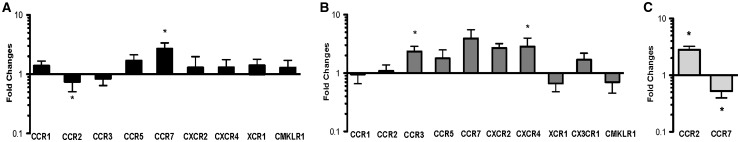
miR-155 regulated chemokine receptor expression Chemokine receptor mRNA expression in PB CD14+ monocytes from (**A**) RA patients (n = 8), (**B**) healthy controls (n = 8) transfected with miR-155 mimic or control mimics. (**C**) CCR2 and CCR7 expression in bone marrow monocytes that were sorted on the basis of CD11b, Ly6C, CD115 and lack of Ly6G expression. The transcript levels of candidate chemokine receptors were normalized to 18S rRNA (house keeping gene) and then calibrated to control mimic**–**transfected cells or transcript levels of wild-type mice. All data are presented as mean (s.e.m.), and statistical significances were evaluated by using the Mann–Whitney test *P ≤ 0.05.

Monocyte trafficking in response to chemokines into tissue sites of inflammation is under the control of specific chemokine receptors [12, 13]. Therefore, we investigated the impact of miR-155 on the expression of chemokine receptors by enforced expressing of miR-155 in CD14^+ ^blood monocytes from RA and healthy subjects. Since chemokine receptors are generally poorly recognized by antibodies, we evaluated their transcriptional regulation herein. Among the 15 chemokine receptors tested, miR-155 transfected into RA CD14^+ ^blood monocytes stimulated an increase in transcript levels of CCR7 and decreased expression of CCR2 (Fig. 4A). Similarly, CCR7 was upregulated in healthy monocytes, although this did not reach statistical significance. In addition, CCR3 and CXCR4 expression was increased in miR-155–transfected healthy monocytes compared with CM-transfected cells (Fig. 4B). Other chemokine receptors, including CCR4, CCR6, CCR8, CCR9 and CCR10 were low or were below the limit of assay detection (CX_3_CR1 in RA patients). Pro-inflammatory imprinting of the RA blood monocytes is likely a contributing factor to the distinct effect of miR-155 on chemokine receptors in RA as compared with healthy blood monocytes.

To validate these data in the context of definitive miR-155 deficiency, we performed a comparative study using *miR-155* gene–deficient mice. Bone marrow monocytes (BMMOs) were sorted based on their expression of CD11b, Ly6C and CD115 and their lack of Ly6G markers (supplementary Fig. S6, available at *Rheumatology* Online). MiR-155^−/−^ BMMOs constitutively expressed a significantly higher level of CCR2 and showed downregulation of CCR7 expression (Fig. 4C). The full range of chemokine receptor expression on miR-155^−/− ^BMMOs is shown in supplementary Fig. S7, available at *Rheumatology* Online).

The contrasting expression of CCR2 and CCR7 between RA blood monocytes with enforced expression of miR-155 and miR-155–deficient mouse monocytes suggests a potential evolutionarily conserved role for miR-155 in regulation of the expression of these receptors.

## Discussion

There is intense interest in unravelling those factors that coordinately regulate cytokine and chemokine production by macrophages in RA synovitis. Prior studies have implicated miR-155 in the regulation of cytokines in macrophages of synovial origin [6, 14]. No studies, however, have addressed the potential role for miR-155 beyond this to the regulation of chemokine or chemokine receptor expression. Moreover, no studies have investigated the role of miR-155 at the level of absolute copy-number in monocytes and its clinical significance for disease activity. We provide herein systematic evidence that miR-155 increased production of the inflammatory chemokines CCL3, CCL4, CCL5 and CCL8, and regulated CCR2 and CCR7 chemokine receptor expression in RA PB monocytes. The combination of these phenotypic manifestations implicates miR-155 in co-ordinating leucocyte recruitment and retention at the joint spaces in inflammatory disease. This is reflected by a tight positive correlation of miR-155 copy-number in PB monocytes with disease activity.

Dynamic regulation of chemokine production and chemokine receptor expression provides a putative evolutionarily conserved molecular mechanism that allows cells to move from one tissue compartment to another. miR-155 copy-number analysis in monocytes revealed the differential expression of miR-155 between blood and the synovial compartment, suggesting an important role for miR-155 in the regulation of this process. High expression levels of miR-155 triggered, at transcriptional and protein levels, increased CCL3 (a ligand for CCR1, CCR3 and CCR5), CCL4 (a ligand for CCR5), CCL5 (a ligand for CCR1 and CCR5) and CCL8 (a ligand for CCR2), which have been asserted as mediating monocyte and T cell recruitment into inflamed joints [15, 16]. This was associated with a reduction in the mRNA expression of inflammatory chemokine receptor and marker of classical monocytes CCR2 [12] in RA CD14^+ ^cells. This downregulation of CCR2 on monocytes after transit into the joint would prevent subsequent egress from the synovial space. A negative regulatory role for miR-155 in the expression of this receptor was supported by the phenotype of miR-155^−^^/^^−^ monocytes, which showed upregulation of many pro-inflammatory chemokine receptors, including CCR2. In contrast to monocytes from RA patients and mouse BMMOs, CCR2 seemed not to be affected by miR-155 in monocytes from healthy donors. We speculate that this could be due to the difference in the composition of CCR2-expressing monocyte subpopulations in the different experimental groups. RA blood contains an increased percentage of CCR2 inflammatory classical monocytes [17], and the Ly6C^high^ monocytes from mouse bone marrow used in this study are also characterized by high CCR2 expression [12]. Thus, miR-155 manipulation might have had an impact only in cells expressing high levels of CCR2.

**Fig. 5:**
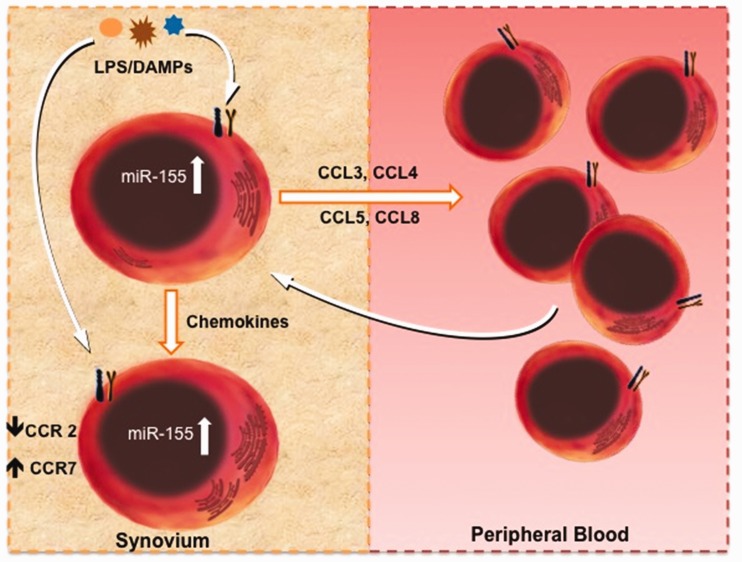
Schematic representation of the role of miR-155 in migration and retention of inflammatory cells in synovial tissue In the RA synovium, Toll-like receptor ligands and a direct contact with activated T cells strongly upregulated miR-155 expression in monocytes/macrophages, and this led to production of pro-inflammatory cytokines and chemokines. Chemokine activity recruited monocytes and other cells from the peripheral circulation into synovial tissue. Simultaneously, high expression of local miR-155 leds to downregulation of CCR2 chemokine receptors on synovial monocytes/macrophages and facilitated their retention in the synovium. Images of the cell were taken from the Protein Lounge (www.proteinlounge.com).

Blood monocytes from active RA appear to be imprinted to disproportionately increase miR-155 expression upon Toll-like receptor 4 (TLR4) engagement. This is commensurate with the highest expression levels of miR-155, which occur in SF CD14^+ ^cells situated in a strongly pro-inflammatory environment (compared with the blood compartment of healthy subjects and RA patients). Thus, we suggest that miR-155 induced by endogenous TLR ligands in the synovial milieu or by contact with other inflammatory cells [6, 18] exists at high copy-number in RA SF monocyte/macrophages and mediates chemokine production that in turn leads to recruitment of blood monocytes and T cells into the joint space; this then attenuates the expression of inflammatory chemokine receptors, retaining these activated cells in the synovium. In addition, our data indicate that CCR7 is positively regulated by miR-155. CCR7 and its corresponding ligands have been implicated in lymphoid neogenesis: they are localized in the lymphocytic infiltration and in dendritic cells (DCs) in the RA synovium and play a crucial role in the maturation and homing of DCs to lymphocytic aggregation [19, 20]. Thus, we speculate that some inflammatory monocytes expressing high levels of miR-155 and CCR7 can give rise to inflammatory DCs that are directed to ectopic lymphoid structures in synovium (Summary Fig. 5).

The mechanism of miR-155 regulation of chemokines and chemokine receptors is currently unknown. It is likely that miR-155 impacts the pro-inflammatory signalling pathways implicated in differential chemokine and chemokine receptor system expression. This could include the signalling pathways mediated by validated miR-155 targets SHIP-1 [21] and SOCS-1 [22], which are inhibitors of myeloid cell activation. In addition, the impact of miR-155 on monocyte function could be influenced by the presence of other post-transcriptional regulators of the inflammatory response, including miR-146 [23] or *lincRNA-*Cox2 [24], which can lead to a variation in the miR-155–mediated chemokine/chemokine receptor expression between healthy subjects and RA patients. Further studies are required to establish the mechanism by which miR-155 regulates the balance between chemokine, cytokine and chemokine receptor expression.

In accord with this observation and with other described pro-inflammatory activities of miR-155, the copy-number of miR-155 in blood-derived monocytes tightly correlated with rheumatoid arthritis clinical markers including DAS28, Total /swollen joint counts and ESR. Future studies will be required to determine whether miR-155 can serve as a useful biomarker of RA disease activity, perhaps included as part of a poly-factorial algorithm. This may be particularly useful for myeloid targeting therapeutics—for example, those targeting the GM-CSF pathway. Several other studies performed on whole PBMCs or serum have emphasized the clinical significance of miRNAs in arthritis as disease-specific biomarkers (serum miR-16 and miR-223 in early RA, and serum miR-24 and miR-125a-5p in established RA) or inflammation-specific biomarkers (miR-146a, miR-132 and miR-16 in PBMCs) [10, 25, 26].

Analysis of the influence of drug therapy on miR-155 copy-number expression revealed no difference between the conventional DMARDs– and the biologics-treated groups. However, due to the small sample size of the biologics group in this study, it is recommended that this be re-evaluated in a larger study appropriately powered for biomarker discovery.

In summary, our data delineated the complex interaction between chemokine-mediated migration, inflammatory factor stimulation and microRNA epigenetic control of monocytes in inflammatory joint disease. Our results collectively imply that miR-155 can act as an important epigenetic regulator of chemokine and chemokine receptor expression and is a key factor in the clinical manifestation of RA and its pathogenesis.

## Supplementary Material

Supplementary Data
