# Supplementary material for: MicroRNA-155 regulates monocyte chemokine and chemokine receptor expression in Rheumatoid Arthritis
Source: Rheumatology (Oxford). 2016 Jul 13;55(11):2056–65. doi: 10.1093/rheumatology/kew272 (PMC5088623; doi:10.1093/rheumatology/kew272)
Supplement: Supplementary Data [file supp_55_11_2056__index.html]

MicroRNA-155 regulates monocyte chemokine and chemokine receptor expression in Rheumatoid Arthritis — Supplementary Data 

# MicroRNA-155 regulates monocyte chemokine and chemokine receptor expression in Rheumatoid Arthritis

## Supplementary Data

files

- Supplementary Data - docx file
