# Supplementary material for: MicroRNA-155 regulates monocyte chemokine and chemokine receptor expression in Rheumatoid Arthritis
Source: Rheumatology (Oxford). 2016 Jul 13;55(11):2056–65. doi: 10.1093/rheumatology/kew272 (PMC5088623; doi:10.1093/rheumatology/kew272)
Supplement: Supplementary Data [file supp_kew272_rhe-15-1391-File007.docx]

Supplementary Table S1. Demographic, clinical and laboratory information on Rheumatoid Arthritis patients who donated PB

| Subject | Age | Sex | DD | ACPA | ESR | CRP | DAS28 | Treatment |
| --- | --- | --- | --- | --- | --- | --- | --- | --- |
| RA1 | 59 | F | 16 | **+** | 22 | 9 | 3.5^a^ | HCQ+MTX+SSZ |
| RA2 | 61 | M | 8 |  | 75 | 46 | 3.85^a^ | MTX |
| RA3 | 62 | F | 13 | **+** | 60 | 11 | 5.16^b^ | LEM |
| RA4 | 73 | F | 33 |  | 12 | 0.5 | 2.89^c^ | MTX+ Adal |
| RA5 | 63 | M | 27 |  | 16 | 8.6 | 4.38^a^ | MTX+ Inflix |
| RA6 | 67 | M | 27 |  | 7 | 3.7 | 2.2^c^ | HCQ+MTX+SSZ |
| RA7 | 70 | F | 15 | **+** | 40 | 12 | 4.6^a^ | MTX+ Etan |
| RA8 | 62 | F | 22 | **+** | 26 | 7 | 3.8^a^ | SSZ |
| RA9 | 53 | M | 10 |  | 38 | 3.3 | 4.7^a^ | SSZ |
| RA10 | 67 | M | 4 |  | 14 | 3 | 3.9^a^ | MTX+ Gold |
| RA11 | 59 | M | 17 | **+** | 19 | 5 | 3.9^a^ | MTX |
| RA12 | 67 | F | 41 | **+** | 31 | 5.2 | 4.36^a^ | LFM+HCQ |
| RA13 | 55 | F | 8 | **+** | 10 | 2.4 | 2.95^c^ | SSZ |
| RA14 | 75 | F | 9 |  | 20 | 5.1 | 2.4^c^ | MTX+ SSZ |
| RA15 | 58 | M | 5 | **+** | 41 | 27 | 5.3^b^ | HCQ+MTX+SSZ |
| RA16 | 71 | F | 10 | **+** | 31 | 1.2 | 3.36 | HCQ+MTX+SSZ |
| RA17 | 70 | F | 13 | **+** | 34 | 10 | 5.2^b^ | MTX+ Adal |
| RA18 | 62 | F | 22 | **+** | 10 | 5 | 2.5^c^ | MTX |
| RA19 | 58 | M | 20 | **+** | 60 | 22 | 5.8^b^ | MTX+ SSZ+ Adal |
| RA20 | 36 | F | 4 | **+** | 30 | 6.6 | 4.6^a^ | MTX+ SSZ |
| RA21 | 63 | F | 30 | **+** | 60 | 11 | 4.8^a^ | SSZ |
| RA22 | 74 | F | 37 | **+** | 40 | 13 | 4.95^a^ | SSZ+HCQ |
| RA23 | 60 | F | 15 | **+** | 12 | 1.6 | 3.2^a^ | MTX+ SSZ+ Inflix |
| RA24 | 76 | F | 21 | **+** | 15 | 5.6 | 4.4^a^ | MTX+ SSZ |

Normal ESR is <20mm/h for female and <10 mm/h for male. Normal CRP is < 4.9mg/l. ^a^moderate disease activity; ^b^high disease activity. ^c^in remission/low disease activity. DD: disease duration; LEM: Leflunomide.

**Supplementary Table S2. Demographic, clinical and laboratory information of Rheumatoid Arthritis patients who donated SF**

| Subject | Sex | Age | ACPA | CRP | ESR | DAS28 | miR-155 Copy No. |
| --- | --- | --- | --- | --- | --- | --- | --- |
| SF1 | F | 35 | + | 13 | 8 | 3.6 | 65200 |
| SF2 | F | 34 | + | 3 | 13 | 3.61 | 44100 |
| SF3 | F | 41 | - | 18 | 28 | 3.85 | 28200 |
| SF4 | F | 48 | + | 0.6 | 12 | 3.48 | 72200 |
| SF5 | F | 41 | + | 2.3 | 5 | 3 | 33400 |
| SF6 | F | 70 | - | 1.4 | 10 | 3.07 | 23700 |
| SF7 | F | 37 | + | 38 | 62 | 4.49 | 154000 |
| SF8 | F | 35 | - | 20 | 26 | 4.02 | 133000 |
| SF9 | F | 42 | - | 14 | 44 | 4.82 | 21700 |
| SF10 | M | 37 | - | 11 | 19 | 3.31 | 36000 |
| SF 11 | F | 78 | + | 15 | 30 | 4.41 | 217000 |

Normal ESR is <20mm/h for female and <10 mm/h for male. Normal CRP is < 4.9mg/l. CRP: C-reactive protein; M: male; F: female.

Supplementary Table S3. Cell purity and transfection efficiency of PB CD14^+^ isolated from both healthy controls and RA patients

| Healthy controls | | | Rheumatoid Arthritis | | |
| --- | --- | --- | --- | --- | --- |
| Subject | CD14^+^ Purity | TE | **Subject** | CD14^+^ Purity | TE |
| HC1 | 97 | 86 | **RA 1** | 99 | 41^a^ |
| HC2 | 97 | 59^a^ | **RA 2** | 94 | 76 |
| HC3 | 95 | 50^a^ | **RA 3** | 97 | 64 |
| HC4 | 97 | 64 | **RA 4** | 93 | 69 |
| HC5 | 97 | 76 | **RA 5** | 92 | 82 |
| HC6 | 96 | 85 | **RA 6** | 90 | 65 |
| HC7 | 96 | 43^a^ | **RA 7** | 90 | 51^a^ |
| HC8 | 98 | 63 | **RA 8** | 92 | 40^a^ |
| HC9 | 98 | 71 | **RA 9** | 93 | 70 |
| HC10 | 98 | 87 | **RA 10** | 94 | 86 |
| HC11 | 97 | 37^a^ | **RA 11** | 97 | 40^a^ |
| HC12 | 98 | 35^a^ | **RA 12** | 97 | 60 |
| HC13 | 98 | 85 | **RA 13** | 96 | 65 |
| HC14 | 95 | 40^a^ | **RA 14** | 97 | 64 |
| HC15 | 90 | 78 | **RA 15** | 94 | 82 |
| HC16 | 93 | 74 | **RA 16** | 90 | 37^a^ |
| HC17 | 93 | 40^a^ | **RA 17** | 92 | 73 |
| HC18 | 98 | 78 | **RA 18** | 98 | 25^a^ |
| HC19 | 93 | 80 | **RA 19** | 85 | 87 |
| HC20 | 98 | 81 | **RA 20** | 90 | 88 |
| HC21 | 92 | 85 | **RA 21** | 92 | 31^a^ |
| HC22 | 90 | 81 | **RA 22** | 94 | 48^a^ |
|  |  |  | **RA 23** | 92 | 82 |
|  |  |  | **RA 24** | 90 | 64 |

Cell purity was determined using flow cytometry analysis. PB CD14^+^ cells were transfected with unlabelled or Dy547 labelled control mimic. Transfection efficiency was calculated as % of cell positive with Dy547. ^a^Experiments with transfection efficiency below 60%) were withdrawn from further analysis. TE: transfection efficiency; HC: healthy control.

**Supplementary Figure S1. PB CD14+ of healthy controls, RA patients and SF CD14+ of RA patients after auto-MACS sorting**


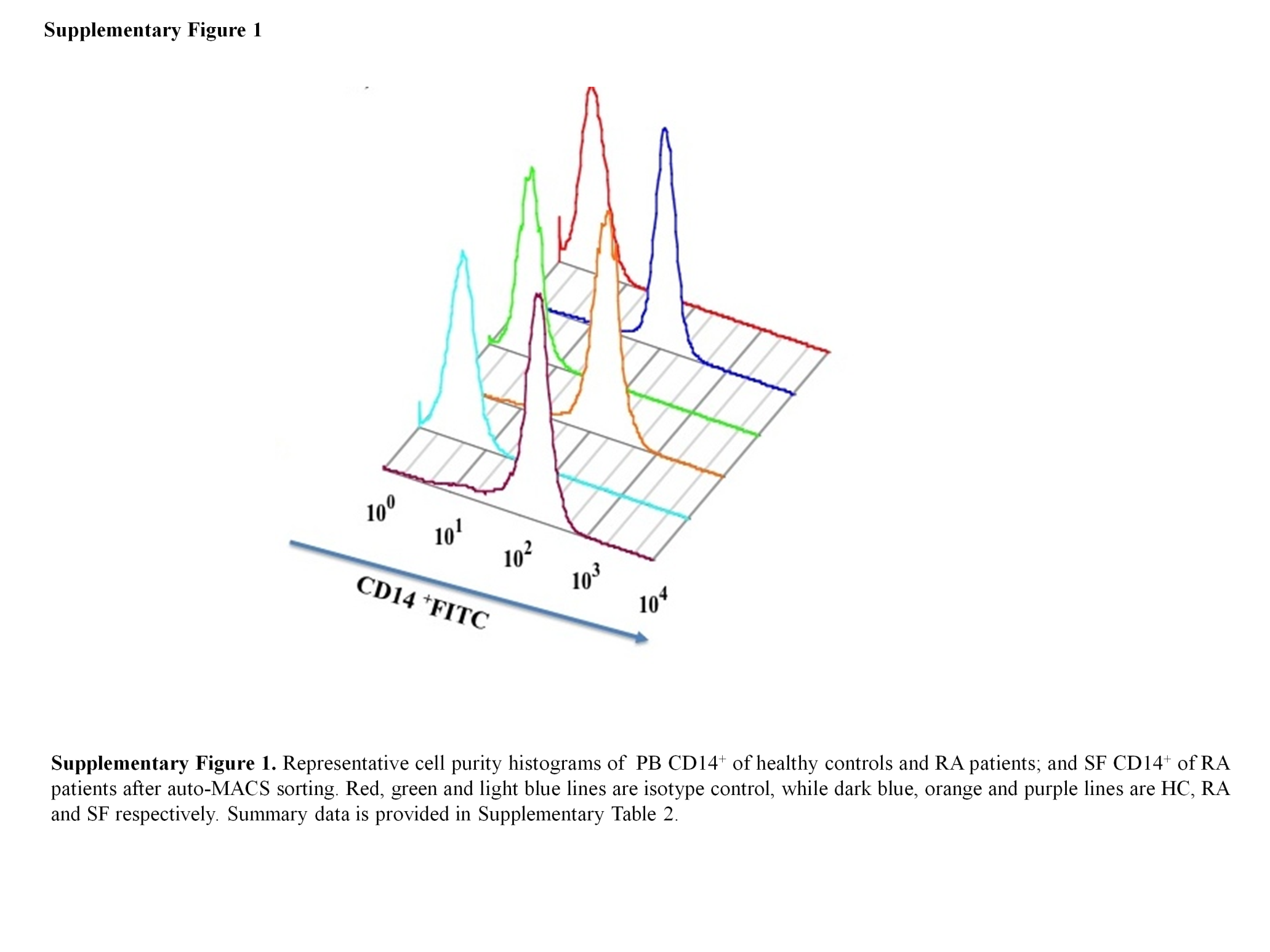


Representative histograms for healthy controls, RA patients and SF CD14+ of RA patients. Red, green and light blue lines are isotype controls, while dark blue, orange and purple lines are HC, RA and SF, respectively. Summary data is provided in Supplementary Table S2. PB: Peripheral blood; MACS: magnetic-activated cell sorting.

**Supplementary Figure S2.** **Transfection efficiency of PB CD14^+^ monocytes with miR-155 mimic**
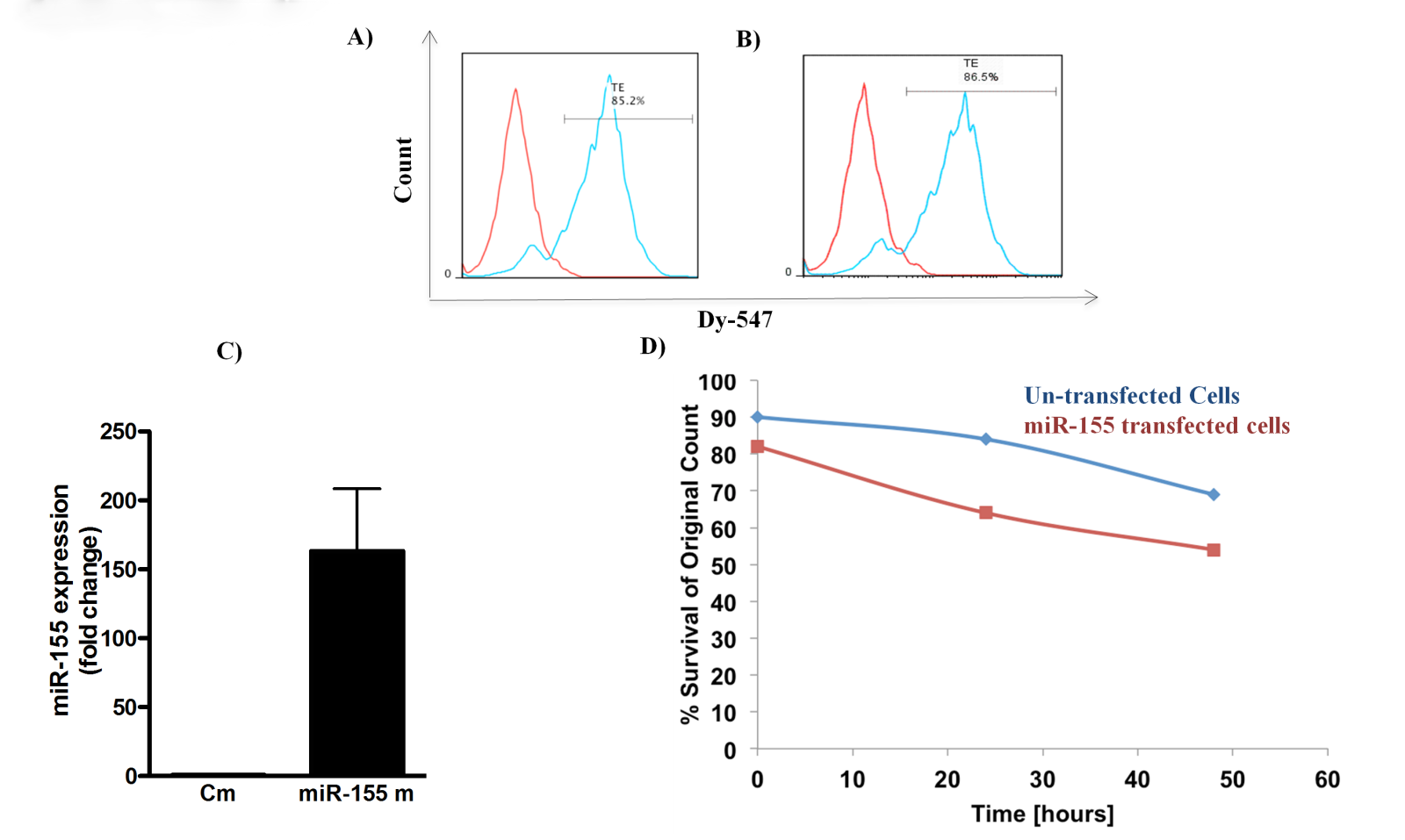


Healthy and RA PB CD14^+^ monocytes were transfected with control mimic: unlabelled or labelled with fluorescence dye Dy547. After 48 h, cells were analyzed by flow cytometry or by quantitative PCR. A) A representative histogram of Dy547 positive monocytes of healthy control. B) A representative histogram of Dy547 positive monocytes of the RA patient. C) Expression of miR-155 normalized to RNU1A is shown. D) A representative graph of CD14^+^ viability after transfection. Cells were counted with trypan blue. TE: transfection efficiency; Cm: control scramble mimic; miR-155m: miR-155 mimic.

**Supplementary Figure S3. Copy numbers of miR-155 transcripts in PB CD14^+^ monocytes in RA patients with different disease activity**


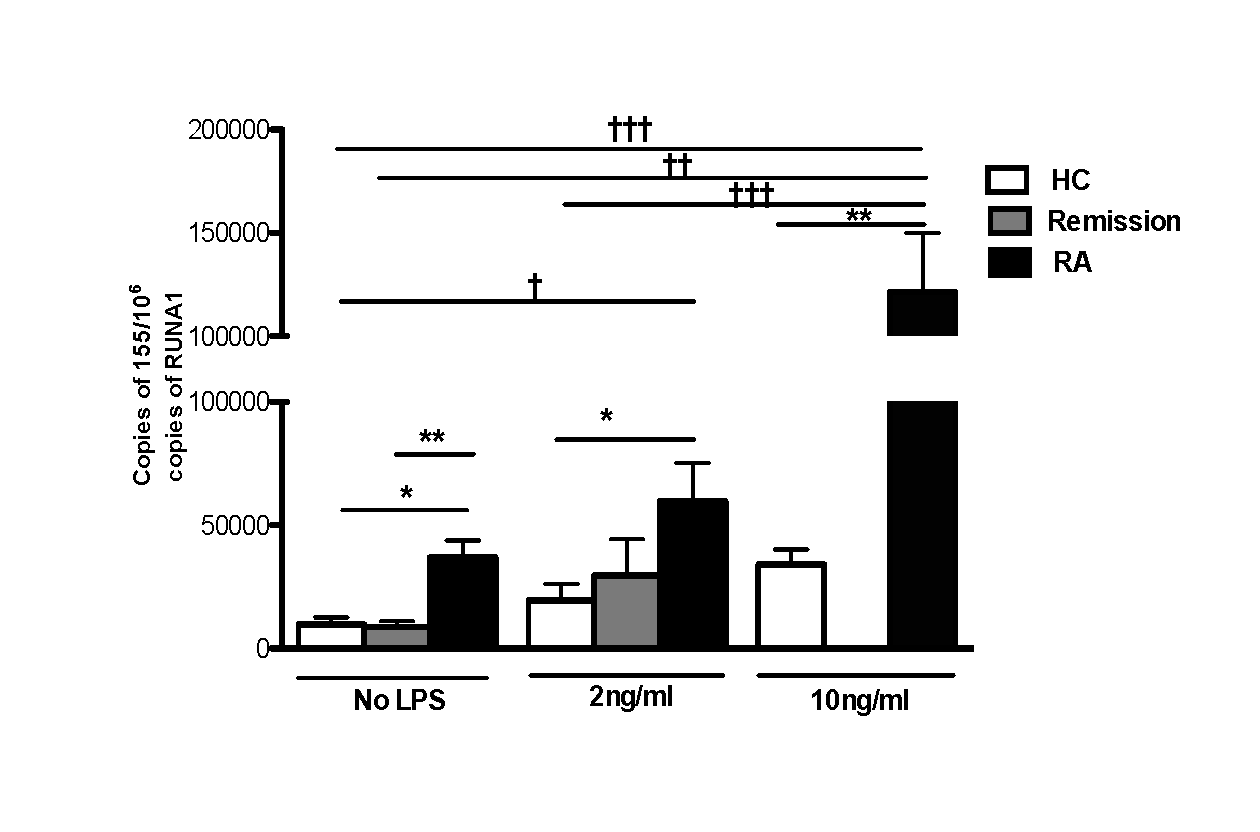


Healthy controls and RA patients cells were cultured in the absence (HC n=22, RA in remission n=5, active RA n=19) or in the presence of different doses of LPS (2 ng/ml; HC n=18, RA in remission n=5, Active RA n=17) or (10 ng/ml; HC n=9, RA in remission n=0, active RA n=16) for 24h. The copy numbers of miR-155 were normalized to 1x106 copies of RNU1A. *HC vs RA (*p≤0.05 and **p≤0.005). †stimulation vs control conditions (†p≤0.05, ††p≤0.005 and †††p≤0.0005). RA in remission DAS28: 2.2-2.95; Active RA DAS28: 3.2-5.8.

**Supplementary Figure S4.** **Cytokine production by PB CD14^+^ monocyte**s
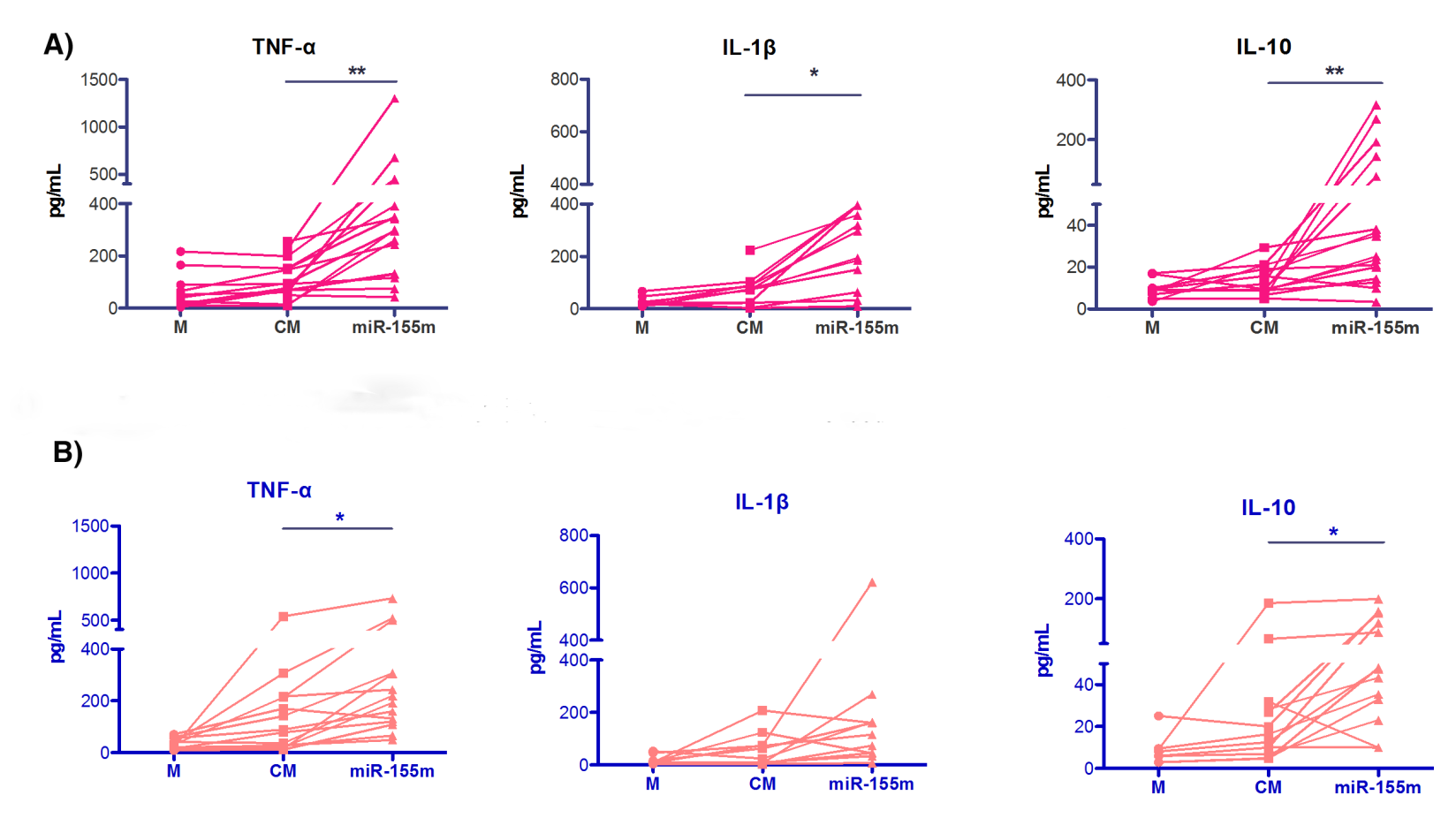


Monocytes from RA patients (A, n=16) B) and healthy donors (B, n=16) were transfected with miR-155 mimic (miR-155m) or control mimic (CM) or left un-transfected (M) are shown. Chemokine levels were measured 48 h later with pre-designed multiplex assay and were tested in triplicate. Values are presented as mean ± SEM. *=p≤ 0.05 and **=p≤ 0.005; Mann-Whitney test or Kruskal-Wallis. Cm, control mimic, miR155m: miR-155 mimic and M; Media.

**Supplementary Figure S5. Cytokine and chemokine mRNA expression in PB CD14**^+^ **monocytes**


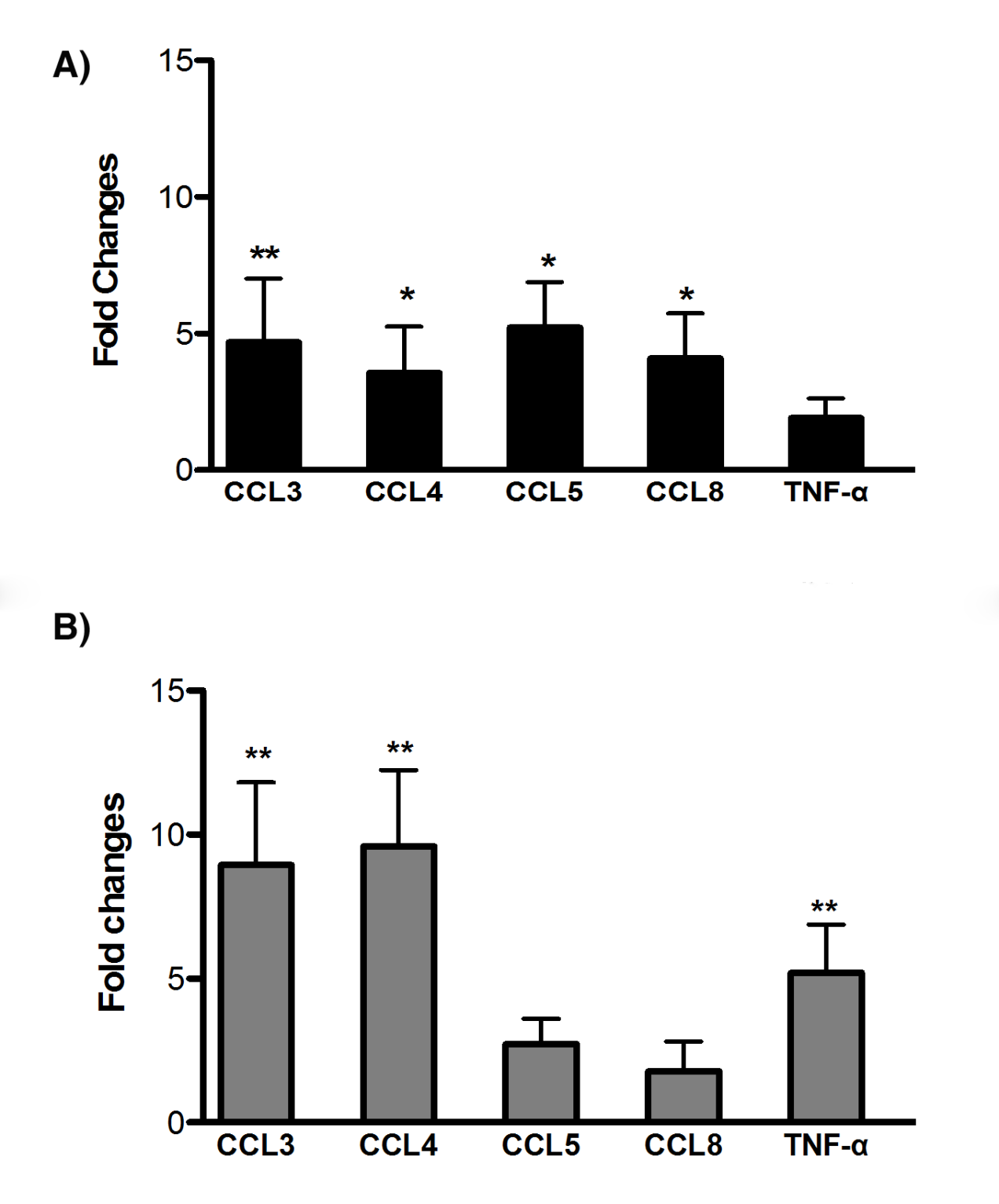


Cells from RA patients (A, n=8) and healthy controls (B, n=8) were transfected with miR-155 mimic or control mimics. After 48h total RNA was extracted and cytokine and chemokine were determined using TLDA plates with specific primers and probes. The transcript levels of candidate genes were normalised to 18S (house keeping gene) and calibrated to control mimic transfected cells then presented as fold changes. Data are presented as mean of 8 patients ± SEM. *=p≤ 0.05, Mann-Whitney test or Kruskal-Wallis test were used to evaluate statistical significances.

**Supplementary Figure S6.** **Gating strategy for purification mouse bone marrow monocytes**


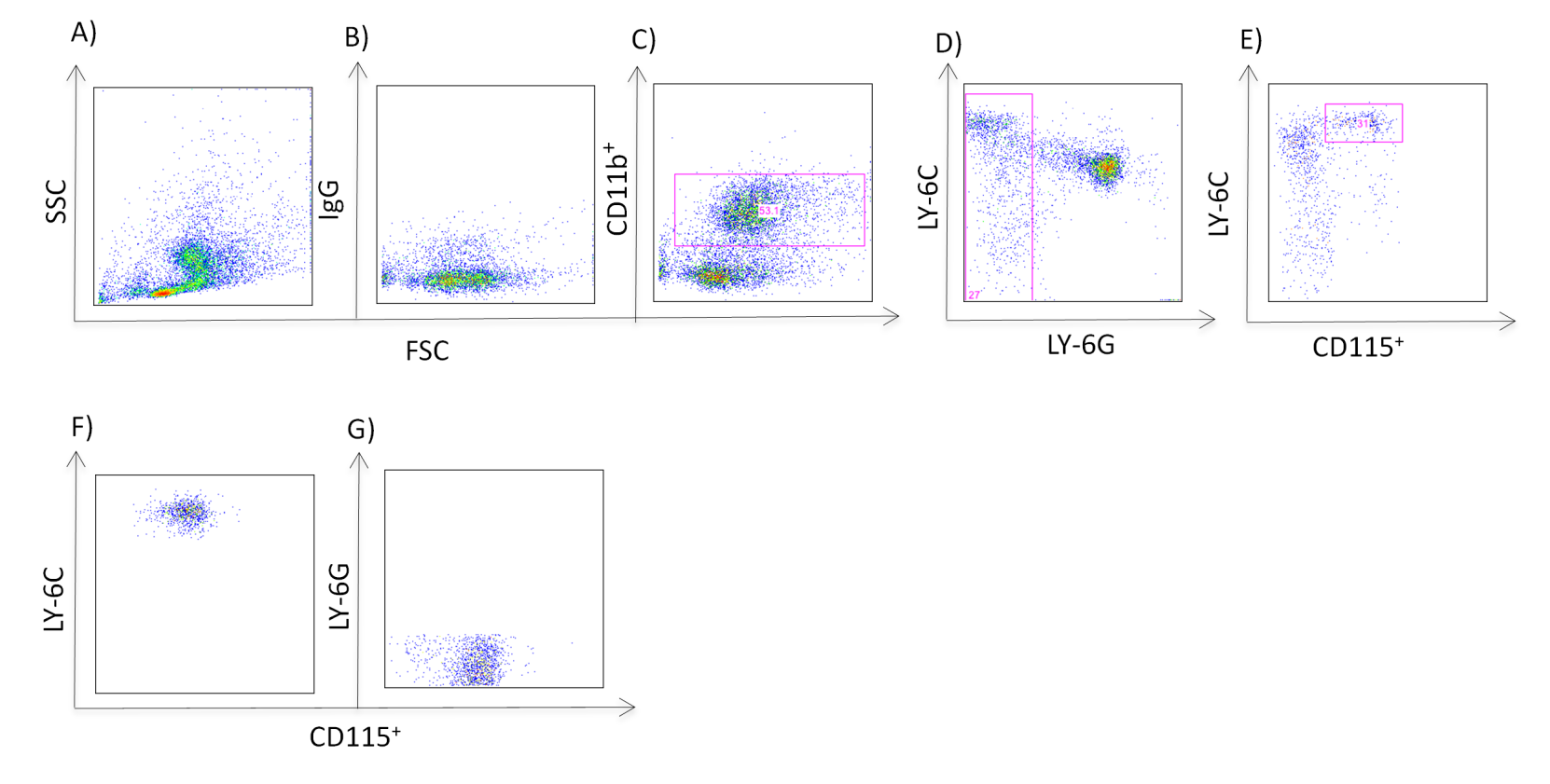


Cells were sorted based on the expression of CD11b, Ly6C and CD115 using a FACS Aria III. Representative dot plots of the whole bone marrow defined by forward scatter (FSC) and side scatter (SSC) parameters (A), cells stained with control IgGs (B) are shown. C) CD11b^+^ expression on whole bone marrow cells demonstrates entire myeloid population. D) Ly6C^+^Ly6G^-^ (monocytes) are gated. E) Monocyte population is refined by the presence of the expression of M-CSFR (CD115). F and G post-sorting purity BMMO (based on their expression of Ly6C and CD115) is shown (98% ± 2%.) Results are representative of 6 experiments.

**Supplementary figure S7.** **Chemokine receptor expression in miR-155^-/-^ bone marrow monocytes**


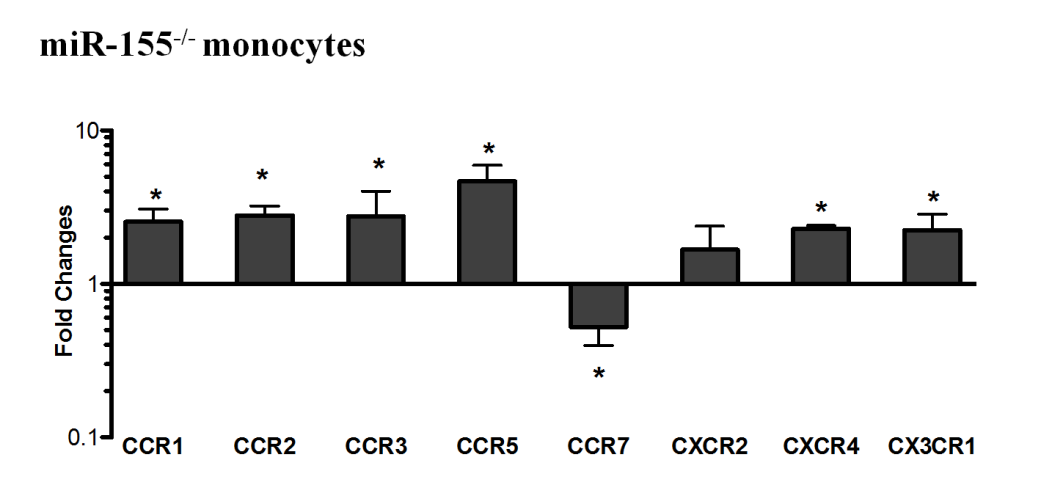


miR-155^-/-^ and wild-type mice that were sorted on the basis of CD11b, Ly6C, CD115 expression and a lack of Ly6G expression. Transcripts levels of chemokine receptors expression in bone marrow monocytes were determined by QPCR using TLDA plates with specific primers and probes. The transcript levels of candidate chemokine receptors were normalised to 18S (housekeeping gene) and then calibrated to transcript levels of WT mice. All data are presented as mean ± SEM and statistical significances were evaluated by using Mann-Whitney test * = p ≤0.05.
